# Gene expression profile of adhesion and extracellular matrix molecules during early stages of skeletal muscle regeneration

**DOI:** 10.1111/jcmm.15624

**Published:** 2020-07-18

**Authors:** Laura C. Ceafalan, Maria Dobre, Elena Milanesi, Andrei M. Niculae, Emilia Manole, Mihaela Gherghiceanu, Mihail E. Hinescu

**Affiliations:** ^1^ Cell Biology, Neurosciences and Experimental Myology Laboratory 'Victor Babeș' National Institute of Pathology Bucharest Romania; ^2^ Department of Cellular and Molecular Biology and Histology Faculty of Medicine ‘Carol Davila’ University of Medicine and Pharmacy Bucharest Romania; ^3^ Molecular Pathology Laboratory ‘Victor Babeș' National Institute of Pathology Bucharest Romania; ^4^ Radiobiology Laboratory ‘Victor Babeș' National Institute of Pathology Bucharest Romania; ^5^ Ultrastructural Pathology Laboratory ‘Victor Babeș' National Institute of Pathology Bucharest Romania

**Keywords:** adhesion molecules, extracellular matrix molecules, gene expression profile, skeletal muscle regeneration

## Abstract

Skeletal muscle regeneration implies the coordination of myogenesis with the recruitment of myeloid cells and extracellular matrix (ECM) remodelling. Currently, there are no specific biomarkers to diagnose the severity and prognosis of muscle lesions. In order to investigate the gene expression profile of extracellular matrix and adhesion molecules, as premises of homo‐ or heterocellular cooperation and milestones for skeletal muscle regeneration, we performed a gene expression analysis for genes involved in cellular cooperation, migration and ECM remodelling in a mouse model of acute crush injury. The results obtained at two early time‐points post‐injury were compared to a GSE5413 data set from two other trauma models. Third day post‐injury, when inflammatory cells invaded, genes associated with cell‐matrix interactions and migration were up‐regulated. After day 5, as myoblast migration and differentiation started, genes for basement membrane constituents were found down‐regulated, whereas genes for ECM molecules, macrophage, myoblast adhesion, and migration receptors were up‐regulated. However, the profile and the induction time varied according to the experimental model, with only few genes being constantly up‐regulated. Gene up‐regulation was higher, delayed and more diverse following more severe trauma. Moreover, one of the most up‐regulated genes was periostin, suggestive for severe muscle damage and unfavourable architecture restoration.

## 1 BACKGROUND

Muscle trauma is a highly frequent event, both accidental and iatrogenic. Despite skeletal muscle having an outstanding regeneration capacity, major injuries often produce persistent pain and functional impairment.^1^ Prevention and management are hindered by the lack of scientific evidence, particularly lack of specific biomarkers to predict severity, progression and prognosis of these injuries.

Skeletal muscle repair after both traumatic and contusion‐induced injury is a complex phenomenon relying on distinct, but cooperative and overlapping processes such as degeneration, inflammation, myofibre regeneration, and remodelling. Altogether, it relies on the cooperation of two major cellular categories: satellite cells (SC)/myoblasts and various types of resident and migrated interstitial cells.^2^ During the first few days after acute injury, the latter are represented mostly by several myeloid cell populations, which are recruited at different time‐points of the regeneration process. Mast cells and neutrophils are the first cells to respond. The second wave of cells attracted at the injury site are macrophages (MCs) whose numbers significantly increase 2 days post‐injury.^3^ After performing their phagocytic role, at around 4 days post‐injury, the pro‐inflammatory MCs suffer a phenotypic switch and become proliferative, anti‐inflammatory MCs^4^ that support myogenesis and myofibre growth. The phenotypic and transcriptional pattern induced by this transition coincides with changes in the expression level of developmentally regulated, myogenic genes. These genes govern different steps of the myogenic process,^5^ from the activation and proliferation of myogenic precursors (MP) to their fusion, differentiation and growth. This sequence of the myogenic process can be easily demonstrated in vitro in the absence of the myeloid population. However, recruited myeloid cells release a large array of soluble molecules^6^, ^7^ and establish direct molecular contacts with resident myogenic cells.^8^, ^9^ This complex mechanism regulates the amplitude and the timing of each step along the myogenic process.

Extracellular matrix (ECM) remodelling is also a key process during inflammation, wound healing, and injury repair^10^ and implies both protein synthesis and degradation. The ECM consists of various structural proteins—diverse collagen types, fibronectin, laminin—as well as non‐structural proteins, such as matricellular proteins. The latter have cellular binding sites for cell‐matrix interactions and diffusible growth factors for creating gradients as guiding cues for cell migration and signalling events during tissue regeneration.^11^ The tight synchronization between interstitial and myogenic cells and ECM remodelling is an essential prerequisite for an efficient regeneration and functional recovery, while evading fibrosis.^4^, ^12^, ^13^ To date, however, no studies have investigated ECM and adhesion molecule‐associated gene expression changes during muscle regeneration, and there exist few data correlating gene profiles and types of injury.

The aims of our study were (a) to investigate the in vivo gene expression profile of ECM and adhesion molecules, as premises of homo‐ or heterocellular cooperation and milestones for skeletal muscle regeneration after acute crushing injury and (b) to identify specific genes related to different types of injury, by comparing the expression profile (for ECM and adhesion molecules) induced by our model (crushing) with previously reported injury models.^14^

Thus, these gene expression profiles were measured at two different time‐points after muscle injury, in the time‐frame when inflammation and degeneration are peaking, and as it switches towards regeneration and remodelling.^15^ Gene expression profile in injured muscles was compared both with the contralateral, non‐injured muscles and uninjured controls. This double comparison allowed us to determine whether the crushing injury led to a systemic reaction which induced changes in gene expression on the contralateral, non‐injured muscle.

## 2 METHODS

### 2.1 Experimental animals

All experiments were conducted on 12‐week‐old *C57BL/6J* male mice (The Jackson Laboratory # 000664), as they are the most commonly used strain for skeletal muscle injury models.^14^, ^16^ The animals included in this study were in perfect health. All mice were kept in individual standard cages at 21‐24°C, with 40%‐60% humidity and a 12‐hour light/dark cycle, in the Animal Facility of Victor Babes National Institute of Pathology. Food and water were provided *ad libitum*. All experiments were approved by the ethics committee of Victor Babes Institute of Pathology (no. 2/29.04.2011) and were performed in compliance with the European Directive 2010/63/EU of the European Parliament and of the Council on the protection of animals used for scientific purposes.

Histopathological evaluation was conducted on 9 (n = 9) animals, three mice at each time‐point: 3rd, 5th and 14th day post‐injury. For the gene expression analysis, a total of 19 (n = 19) male mice were used in this experiment. The animals were divided into three groups: 10 mice were used for generating the muscle injury model, 5 for each time‐point, and 9 mice were used as non‐injured, external controls.

### 2.2 Muscle injury model

The *C57BL/6J* mice were anaesthetized by intramuscular injection of 100 mg/kg Ketamine (Kepro BV) in PBS, in the anterior left leg prior to manipulations, before inducing the injury in the posterior left leg and again before being euthanized by cervical dislocation. The level of anaesthesia was assessed by absence of reflexes. The muscle injury model was obtained by crushing the posterior left leg with an adjusted forceps, 1 cm away from the distal joint without fracturing the bone. The pressure was maintained for 2 min. Samples from the injured area of the crushed muscle and from the contralateral gastrocnemius were collected the 3rd and 5th day post‐injury, after cervical dislocation while under anaesthesia, when response to stimuli was no longer detected. These time‐points correspond to the peak of the inflammatory and degeneration stage dominated by inflammatory macrophage recruitment in the injured area, and the switch towards regeneration and remodelling after injury.^17^ Samples from the 14th day post‐injury were also collected for the histopathological assessment of the injury model.

### 2.3 Histopathology

Histopathological evaluation was conducted on three mice at each time‐point: 3rd, 5th and 14th day post‐injury. Small fragments from the left gastrocnemius muscles were collected and fixed by immersion in 4% glutaraldehyde, post‐fixed in buffered 1% OsO_4_ with 1.5% K_4_Fe(CN)_6_ (potassium ferrocyanide–reduced osmium), dehydrated in graded ethanol series and further processed for epoxy resin embedding (AGAR 100). One‐micrometre‐thick sections (semi‐thin sections) were stained with 1% toluidine blue and examined by light microscopy for morphological analysis with Leica DM 600. Images were recorded using a Leica DFC7000 T camera.

### 2.4 RNA isolation and gene expression analysis

Gene expression analysis was performed on total RNA isolated from five pair tissue samples from injured (I) and non‐injured contralateral (N‐I) muscles at 3 and 5 days post‐injury. N‐I was used as internal control. Another external control group (C) of 9 animals (without muscle injury) was enrolled in the study, and 3 pools of RNA were analysed for gene expression. Total RNA isolation was performed using RNeasy Mini Kit (Qiagen) from fresh‐frozen tissues preserved in RNA later, according to the manufacturer's protocols. The quantity and quality of RNA were determined using the Nanodrop 2000 (Thermo Scientific). An amount of 180 ng of RNA was reverse transcribed to cDNA using the RT2 First Strand Kit (Qiagen). The Mouse Extracellular Matrix & Adhesion Molecules RT2 Profiler PCR Array (PAMM‐013Z, Qiagen) using SYBR Green chemistry was used to evaluate the expression of 84 genes, according to the manufacturer's protocol, on the ABI‐7500 fast instrument (Applied Biosystems). The stability of five potential reference genes included in the array (ACTB, B2M, GAPDH, GUSB and HSP90AB1) was evaluated with RefFinder, a web‐based program including multiple algorithm methods (http://www.leonxie.com/referencegene.php). Accordingly, the expression levels of each gene were normalized on the geometric mean values of B2M and HSP90AB1.

### 2.5 Gene ontology and pathway analyses

Gene ontology and pathway analyses on the differentially expressed genes (both at 3 and 5 days post‐injury) were performed by GO Molecular Function 2018 and KEGG 2019 Mouse through Enrichr, a comprehensive gene set enrichment analysis web server (PMID: 27141961).

### 2.6 GEO data mining

A search of the NCBI Gene Expression Omnibus (GEO) was conducted in order to find data sets reporting gene expression changes in the skeletal muscle tissue of C57BL/6J mice following different methods of injury. Only GSE5413 was identified.^14^ This data set reports gene expression in the skeletal muscles of uninjured and of injured mice at different time‐points (6 hour, 1, 3 and 7 days), following eccentric contraction injury (CI) or freezing injury (FI), and using the Affymetrix Murine Genome U74A Version 2 Array. The candidate genes were analysed for differential expression using GEO2R in the following comparisons: 3 and 7 days (both CI and FI) vs non‐injured, external controls.

### 2.7 Statistical analysis

Gene expression analysis was conducted using the Statistical Package for Social Science (SPSS Version 17.0). Data normality was assessed using the Shapiro‐Wilk test. Since data were normally distributed (*P* > .05), a paired *t* test was used to assess differences in gene expression levels between I and N‐I (at 3 and 5 days). Comparisons of gene expression levels between I as well as N‐I (at 3 and 5 days) and C were tested with an independent sample *t* test. Difference in gene expression was considered significant when *P* < .05 and fold regulation (FR) > |1.5|.

## 3 RESULTS

### 3.1 Histopathologic evaluation

**Figure 1:**
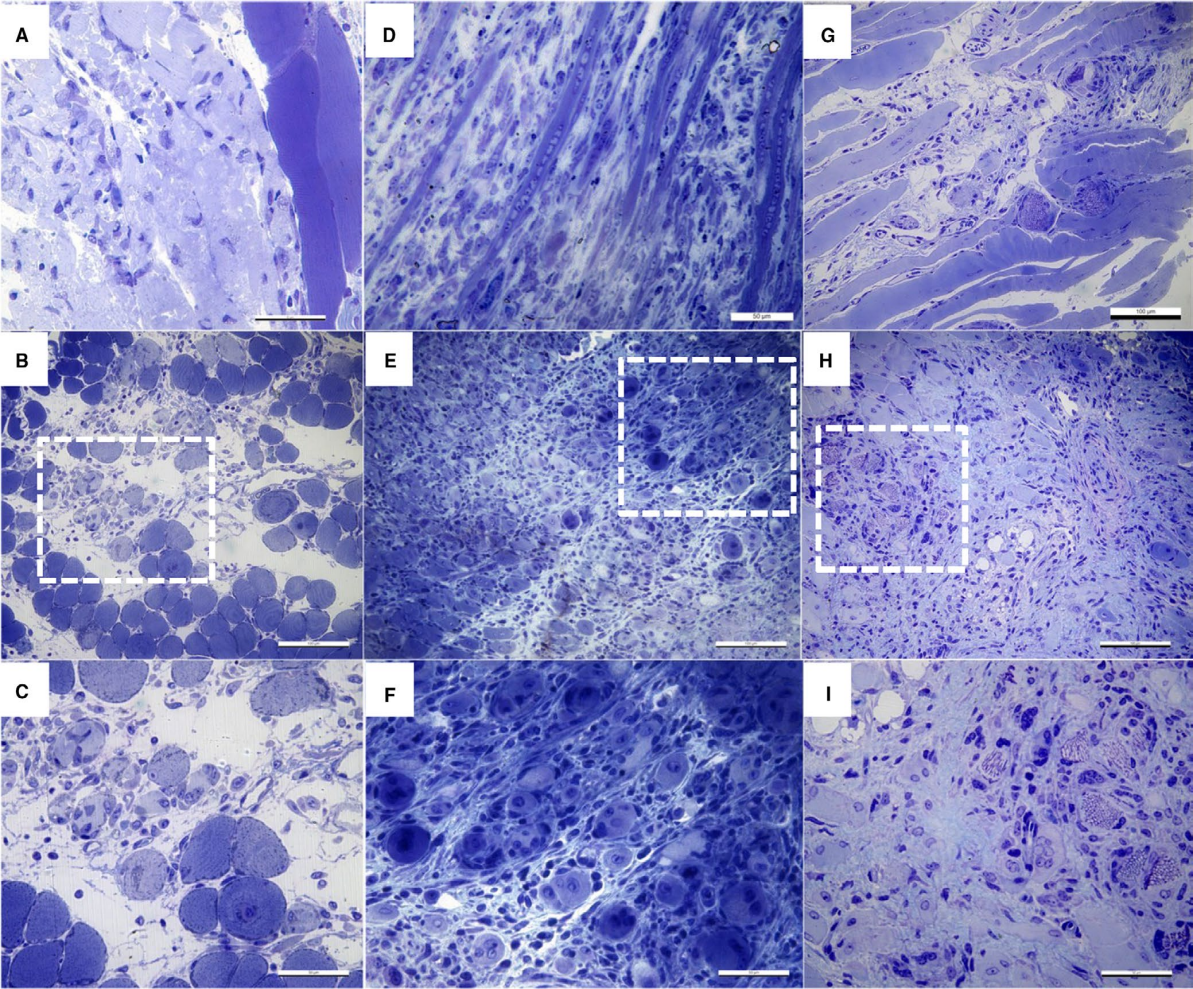
Light microscopy on toluidine blue–stained semi‐thin sections of epoxy‐embedded injured gastrocnemius muscle. Representative images from three different mice. A‐C. 3 days post‐injury oedema and some necrotic fibres are observed along with a massive inflammatory infiltrate in the interstitial spaces around damaged fibres. D‐F. 5 days post‐injury, regenerating myofibres (myotubes with central nuclei) and inflammatory infiltrate are observed. In some areas, muscle necrosis is still present. G‐I. 14 days post‐injury, inflammatory infiltrate and collagen deposition were still detected at the injury site. A, D, G longitudinal sections; B, E, H cross sections; boxed areas are presented at a higher magnification in C, F and I, respectively

Histologic evaluation of the crush injury model was performed on 9 otherwise healthy animals, 3 for each time‐point. Samples harvested 3 days after mechanical trauma showed the presence of oedema and a massive inflammatory infiltrate restricted to the interstitium around degenerated and necrotic muscle fibres (Figure 1A‐C). At 5 days after inflicting the injury, the first signs of the regeneration process were observed, specifically numerous small muscle fibres with central nuclei (myotubes), as well as a decline in the inflammatory infiltrate (Figure 1D‐F). However, the inflammatory infiltrate was still detected even at 14 days post‐injury, along with centrally nucleated myofibres and collagen deposition (Figure 1G‐I).

### 3.2 Gene expression analysis

#### 3.2.1 Gene expression alterations induced by muscle crushing 3 and 5 days post‐injury

##### Injured muscle vs contralateral (I vs N‐I)

The paired analysis (I vs NI) revealed that at 3 days, 9 genes out of 84 were differentially expressed in injured muscle (4 up‐regulated and 5 down‐regulated). Fibronectin 1 (FN1), two genes for cell adhesion matricellular proteins, (THBS2 and VCAN) and one for a transmembrane receptor (ITGAM) were up‐regulated. Surprisingly, no matrix metalloproteinases were significantly up‐regulated, but the expression of MMP‐15, as well as tissue inhibitor of metalloproteinase 3, TIMP3, were found decreased as compared to the internal control at the same time‐point.

When considering the 5‐day group, 19 genes were differentially expressed in the paired analysis: 14 were up‐regulated and another five down‐regulated. Among the up‐regulated genes, most were ECM transcripts of alpha chain fibrillary collagen (III, V, VI) and cell adhesion molecules, both receptors for cell‐to‐ECM adhesion (ITGAL and ITGAX) and matricellular proteins (THBS2, 3, VCAN and POSTN). Also, protease‐associated transcripts started to be up‐regulated at this time‐point. However, transcripts for basement membrane proteins were down‐regulated (Col4A2, LAMA2). POSTN was the most up‐regulated gene in our injury model, with a late induction point.

**Figure 2:**
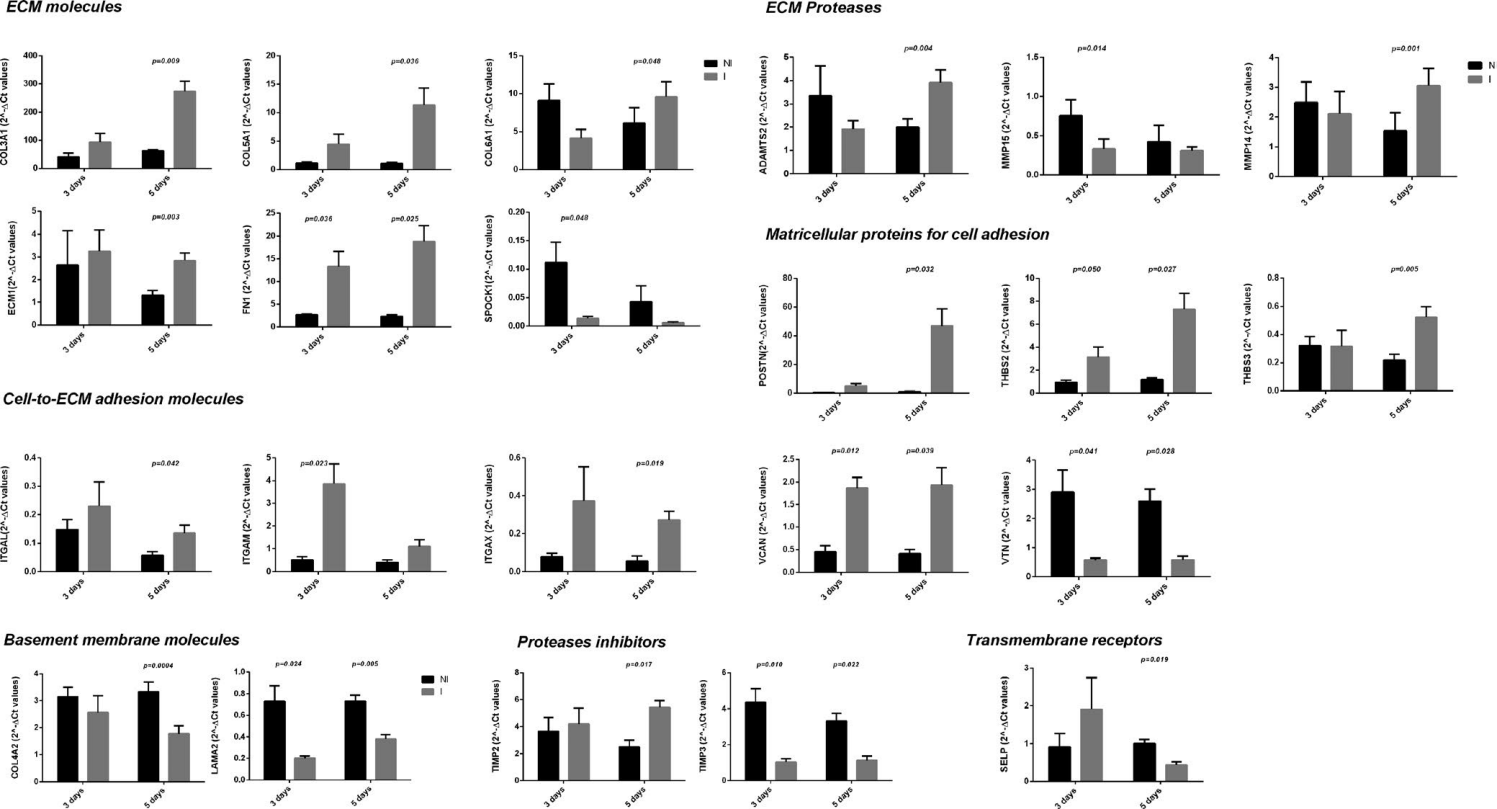
Genes differentially expressed in injured (I) vs non‐injured (N‐I) muscle. Histogram bars represent the level of gene expression as the mean of 2^−∆CT^ values

Only 3 genes showed increased expression both at 3 and 5 days (FN1, THBS2 and VCAN), whereas 3 others showed decreased expression (LAMA2, TIMP3 and VTN) (Figure 2).

#### 3.2.2 Non‐injured muscle (N‐I) and the injured muscle (I) vs external control group (C)

To exclude any systemic influence that might have induced a change in gene expression, we compared genes that were significantly regulated (presented in detail in Table 1) in the N‐I at 3 days and 5 days, versus C. None of the transcripts of interest were differentially expressed (*P* < .05). When considering all 84 genes, it was revealed that Col4A2 was up‐regulated in the N‐I, contralateral muscle as compared to C, both at 3 and 5 days (FR 2.69, p 0.04; FR 2.85, p 0.042, respectively). At 3 days, the N‐I showed an increase of MMP‐8 expression (FR 5.16, *P* = .039), whereas at 5 days the increase was observed for Cdh1 and Selp (FR 1.78, p 0.044; FR 9.12, p 0.002, respectively; File S1).

**Table 1:** Genes differentially expressed in skeletal muscles following crush muscle injury. The table presents transcripts with a FR > |1.5| with *P* < .05 in I vs N‐I and I vs C. Bold fonts indicate genes differentially expressed both at 3 and 5 days

Gene symbol	Gene description	3 days				5 days			
		Paired I vs N‐I		I vs C		Paired I vs N‐I		I vs C	
		FR	P‐value	FR	P‐value	FR	P‐value	FR	P‐value
*ECM molecules*									
COL3A1	Collagen Type III Alpha 1 Chain					4.36	.009	3.76	.033
COL5A1	Collagen Type V Alpha 1 Chain					10.45	.036		
COL6A1	Collagen Type VI Alpha 1 Chain					1.56	.048		
ECM1	Extracellular matrix protein 1					2.16	.003		
**FN1**	**Fibronectin 1**	**4.93**	**.036**			**7.97**	**.025**		
SPOCK1	SPARC/Osteonectin, Cwcv And Kazal Like Domains Proteoglycan 1	−8.13	.048						
Basement membrane molecules									
COL4A2	Collagen Type IV Alpha 2 Chain					**−1.87**	.0004		
**LAMA2**	**Laminin Subunit Alpha 2**	**−3.64**	**.024**			**−1.92**	**.005**		
LAMB2	Laminin Subunit Beta 2			−1.96	.019			−2.79	.011
LAMC1	Laminin Subunit Gamma 1			−1.69	.007			−2.43	.003
Cell‐to‐ECM Adhesion molecules									
ITGAL	Integrin Subunit Alpha L					2.36	.042		
ITGAM	Integrin Subunit Alpha M	7.61	.023	14.92	.022				
ITGAX	Integrin alpha X					4.96	.019	2.84	.048
ITGB2	Integrin Subunit Beta 2			5.26	.047				
CD44	CD44 Molecule			*8.06*	*.015*			4.83	.050
ECM Proteases									
MMP8	Matrix Metallopeptidase 8			16,491	.037			4.919	.038
MMP14	Matrix Metallopeptidase 14					**1.98**	**.001**		
**ADAMTS2**	**A disintegrin‐like and metallopeptidase (reprolysin type) with thrombospondin type 1 motif, 2**					**1.96**	**.004**	**3.16**	**.039**
**MMP15**	**Matrix Metallopeptidase 15**	**−2.28**	**.014**						
Matricellular proteins for cell adhesion									
POSTN	Periostin, osteoblast‐specific factor					**43.39**	**.032**	16.15	.027
**THBS2**	**Thrombospondin 2**	**3.31**	**.050**			**6.17**	**.027**		
THBS3	Thrombospondin 3					**2.4**	**.005**		
**VCAN**	**Versican**	**4.10**	**.012**	**6.95**	**.004**	**4.68**	**.039**	**7.20**	**.019**
**VTN**	**Vitronectin**	**−5.16**	**.041**			**−4.49**	**.028**		
Proteases inhibitors									
TIMP2	TIMP Metallopeptidase Inhibitor 2					**2.1**	**.017**		
TIMP1	TIMP Metallopeptidase Inhibitor 1			5.04	.00001				
**TIMP3**	**TIMP Metallopeptidase Inhibitor 3**	**−4.27**	**.010**			**−2.98**	**.022**		
Transmembrane receptors									
SELP	Selectin, platelet					***−2.33***	***.019***		
SYT1	Synaptotagmin 1							*−2.10*	*.042*

Bold fonts indicate genes differentially expressed both at 3 and 5 days.

Red fonts indicate the up‐regulated genes.

Blue fonts indicated the down‐regulated genes.

A comparison between I and C was performed to evaluate results against those reported in GSE5413. The results of all the comparisons including I vs C are reported in File S2 (Additional Materials). Significant results are presented in Table 1.

#### 3.2.3 Gene ontology and pathway analyses

The results of the gene ontology and KEGG pathway are shown in Table 2a and b. Most relate to peptidase activity, modulators, as well as integrin‐mediated cell‐ECM interaction for migration and signalling.

**Table 2:** (a‐b). Gene ontology and pathway analyses on the differentially expressed genes (both at 3 and 5 days post‐injury) have been performed by GO Molecular Function 2018 (a) and KEGG 2019 Mouse (b) through Enrichr web server

Gene ontology molecular function	Adj *P*‐value	Genes
a		
Metalloendopeptidase inhibitor activity (GO:0008191)	2.01E‐06	TIMP2;TIMP3;SPOCK1;TIMP1
Protease binding (GO:0002020)	9.08E‐06	ECM1;COL3A1;TIMP2;FN1;TIMP3;TIMP1
Platelet‐derived growth factor binding (GO:0048407)	1.72E‐04	COL3A1;COL5A1;COL6A1
Metalloendopeptidase activity (GO:0004222)	6.53E‐04	ADAMTS2;MMP14;MMP15;MMP8
Integrin binding (GO:0005178)	0.0023	VTN;COL3A1;COL5A1;FN1
Metallopeptidase activity (GO:0008237)	0.0026	ADAMTS2;MMP14;MMP15;MMP8
Endopeptidase inhibitor activity (GO:0004866)	0.0031	TIMP2;SPOCK1;TIMP3;TIMP1
Metalloaminopeptidase activity (GO:0070006)	0.0392	MMP14;MMP15
Hyaluronic acid binding (GO:0005540)	0.0391	VCAN;CD44
Metal ion binding (GO:0046872)	0.0454	SELP;POSTN;SYT1;SPOCK1;THBS3

KEGG pathway	Adj *P*‐value	Genes
b		
ECM‐receptor interaction	4.92E‐15	VTN;LAMA2;COL4A2;LAMB2;COL6A1;FN1;LAMC1;THBS2;CD44;THBS3
Focal adhesion	6.79E‐10	VTN;LAMA2;COL4A2;LAMB2;COL6A1;FN1;LAMC1;THBS2;THBS3
PI3K‐Akt signalling pathway	9.21E‐08	VTN;LAMA2;COL4A2;LAMB2;COL6A1;FN1;LAMC1;THBS2;THBS3
Cell adhesion molecules (CAMs)	1.59E‐04	SELP;VCAN;ITGAM;ITGB2;ITGAL
Complement and coagulation cascades	2.57E‐04	VTN;ITGAM;ITGB2;ITGAX
Protein digestion and absorption	2.53E‐04	COL3A1;COL5A1;COL4A2;COL6A1
Regulation of actin cytoskeleton	3.48E‐04	ITGAM;ITGB2;ITGAX;FN1;ITGAL
Leukocyte transendothelial migration	0.0102	ITGAM;ITGB2;ITGAL
Rap1 signalling pathway	0.0463	ITGAM;ITGB2;ITGAL

#### 3.2.4 Gene expression comparison with eccentric contraction‐induced muscle injury (CI) and freeze‐induced muscle injury (FI) models

The GSE5413 data set reports the gene expression of 12 488 genes in C57BL/6J uninjured (control) and injured mice, using eccentric contraction (CI) and freezing injury (FI) models. In order to compare data from our model of crushing injury with those obtained by GSE5413, we had to compare the gene expression profile of I at 3 and 5 days with that of C (Table 1).

**Figure 3:**
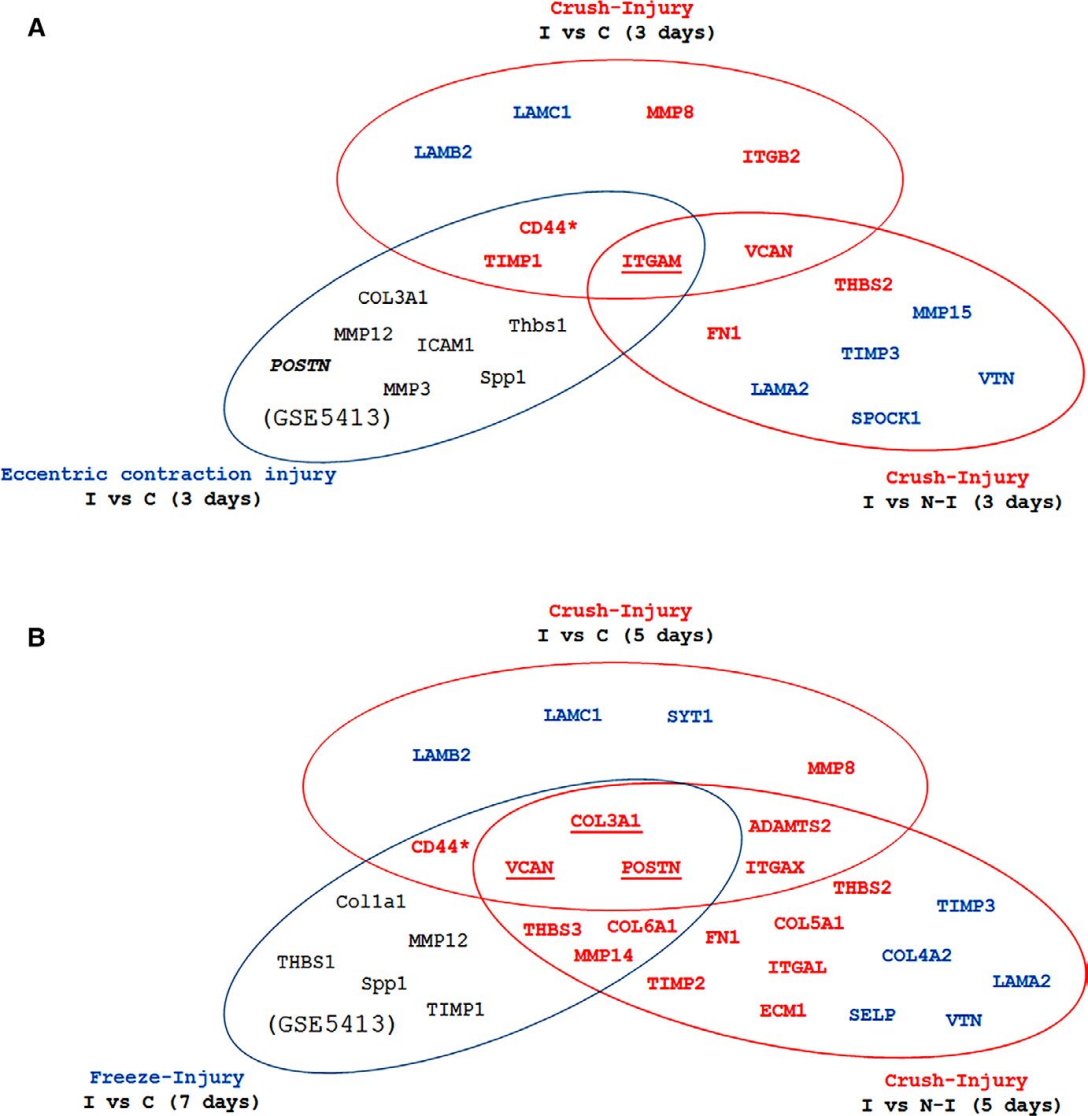
Venn diagram of the relations between gene profiles at 3 days post‐injury (A) and 5/7 days post‐injury (B). Two sets contain the genes differentially expressed in crushed muscle samples compared to contralateral, non‐injured muscle (I vs N‐I) or samples from control, non‐injured animals (I vs C). The third set contains genes differentially expressed in muscle samples after eccentric contraction or freezing injury compared to samples from control, non‐injured animals

GEO data mining found that 10 transcripts out of the 84 we tested were significantly regulated at 3 days post‐injury in the CI model. ITGAM, CD44 and TIMP1 were found up‐regulated in both our model (I vs C) and CI model. Of note, ITGAM was up‐regulated also in the paired analysis (Table 3 and Figure 3A).

**Table 3:** Genes differentially expressed both in crush injury and CI at 3 days after injury

Gene symbol	Gene description	3 days Crush Injury				3 days CI	
		paired I vs N‐I		I vs C		I vs C	
		FR	*P*‐value	FR	*P*‐value	Log2FC	adj *P*‐value
CD44	CD44 Molecule			8.06	.015	2.59	.043
ITGAM	Integrin Subunit Alpha M	7.61	.023	14.92	.022	2.23	.028
TIMP1	TIMP Metallopeptidase Inhibitor 1			5.04	.00001	2.27	.03

Bold fonts indicate genes differentially expressed both at 3 and 5 days.

Red fonts indicate the up‐regulated genes.

Blue fonts indicated the down‐regulated genes.

In the FI model, 12 transcripts out of the 84 we tested were significantly changed at 7 days after inflicting the injury. The expression levels of four genes were significantly increased at 5 days in our own model (I vs C) but also at 7 days in FI model (CD 44, COL3A1, VCAN and POSTN). Three other genes were found commonly up‐regulated in I vs N‐I and FI comparisons (Table 4 and Figure 3B).

**Table 4:** Genes differentially expressed both in crush injury and FI model at 5 and 7 days post‐injury, respectively

Gene symbol	Gene description	5 days Crush Injury				7 days FI	
		paired I vs N‐I		I vs C		I vs C	
		FR	*P*‐value	FR	*P*‐value	Log2FC	adj *P*‐value
CD44	CD44 Molecule			*4.83*	*.050*	*3.36*	*.053* *
*COL3A1*	*Collagen Type III Alpha 1 Chain*	*4.36*	*.009*	*3.76*	*.033*	*4.76*	*.022*
*COL6A1*	*Collagen Type VI Alpha 1 Chain*	*1.56*	*.048*			*2.07*	*.03*
*MMP14*	*Matrix Metallopeptidase 14*	*1.98*	*.001*			*2.37*	*.027*
*POSTN*	*Periostin, osteoblast‐specific factor*	*43.39*	*.032*	*16.15*	*.027*	*5.73*	*.011*
*THBS3*	*Thrombospondin 3*	*2.4*	*.005*			*2.39*	*.03*
*VCAN*	*Versican*	*4.68*	*.039*	*7.20*	*.019*	*2.27*	*.022*

Bold fonts indicate genes differentially expressed both at 3 and 5 days.

Red fonts indicate the up‐regulated genes.

Blue fonts indicated the down‐regulated genes.

* Trend towards statistical significance.

Results of the GSE5413 analysis are reported in File S3 (Additional Materials).

## 4 DISCUSSION

The understanding of the cellular response and molecular composition of the microenvironment during muscle regeneration is mandatory for the development of clinical strategies to improve muscle function during aging, or after extensive trauma. In this study, we evaluated the gene expression profile of ECM and adhesion molecules in skeletal muscle regeneration after acute crush injury and by comparison with other previously reported injury models, such as CI and FI.

Our experimental procedure inflicted direct and severe muscle damage. The histopathology assessment showed collagen deposition and persistent inflammation even at 14 days post‐injury, longer than in the case of small contusion injuries^18^ and other previously reported injury models, like cardiotoxin injection,^19^ CI or even FI.^14^, ^18^ In our model, inflammation gradually resolved and the architecture of the injured muscle was re‐established only after day 21 post‐injury (data not shown).

Few genes coding for adhesion molecules were up‐regulated in the early days of muscle regeneration, the stage when injured area is infiltrated by activated inflammatory myeloid cells. ITGAM (CD11b) up‐regulation is most probably a hallmark of the growing macrophage population through proliferation of resident cells^20^ and further recruitment of activated circulating monocytes. Surprisingly, we found not just that matrix metalloproteinase genes were not up‐regulated during this early phase, but that the expression of MMP‐15 (a membrane‐type metalloproteinase) was transiently decreased as compared to the internal control. MMPs are released from damaged muscle and interstitial cells. Through their enzymatic activity, the various MMPs disrupt the basement membrane of muscle fibres, facilitating the recruitment of SCs and the migration of infiltrating myeloid and interstitial cells.^13^

At 5 days post‐injury, another two metalloproteinases, MMP14 (a membrane‐bound collagenase^21^) and ADAMTS2 (a procollagen processing enzyme), were found to be up‐regulated. However, when compared with the external control, the only change in MMP gene expression is an early, strong and persistent up‐regulation of MMP8 (neutrophil collagenase) which was previously demonstrated to be released from the migrated myeloid population, and to enhance myoblast migration.^22^

Moreover, the gene expression profile for basement membrane molecules suggests a persistently decreased production of both collagen IV and various laminin subunits, when compared with both internal and external controls. This may further facilitate the migration of SCs to the injury site.

Besides facilitating myoblast migration and the angiogenic process,^22^ in vitro studies showed that MMP‐14 regulates myotubes formation by degrading interstitial ECM components, like fibronectin, that prevent cell fusion and laminin alpha 2, regulating the interaction of the mature myofibre with the BM.^23^ In the time‐frame of our experimental model and when compared with the internal control, the up‐regulation of MMP‐14 at 5 days post‐injury follows the early increase of FN1. The concomitant up‐regulation of TIMP‐2 was previously reported as essential to myoblast differentiation.^24^ MMP activity and dynamics are tightly controlled by tissue inhibitors of matrix metalloproteinases (TIMPs), and their expression pattern depends on the type of muscle and the phase of muscle regeneration.^25^ Disturbance in the MMP‐TIMP balance may modulate ECM fibre deposition and further enhance inflammation. In our experimental model, MMP‐8 up‐regulation was accompanied by an early increase in TIMP‐1, but with persistent MMP‐8 up‐regulation at 5 days post‐injury.

The limited and late induction of other MMPs and TIMPs as compared to the GSE5413 data set could be correlated to the prolonged inflammatory response and matrix remodelling observed in our injury model.

Among the up‐regulated genes at 5 days post‐injury, most are transcripts for ECM molecules and a different set of transmembrane receptors for adhesion. Both ITGAX (CD11c) and ITGAL (CD11a) were reported to be expressed by recruited macrophages.^20^ ITGAL/CD11a was previously suggested as part of a survival mechanism based on heterocellular interactions between myoblasts and recruited macrophages.^9^

Only 6 genes were found differentially expressed both at 3 and 5 days post‐injury. One small group of genes comprising FN1, THBS2 and VCAN were constantly up‐regulated. Such genes are usually expressed by fibroblasts but also by activated macrophages^26^ and even differentiating myoblasts.^27^ This group of transcripts encodes for proteins that play important roles in the cell‐matrix interactions during adhesion and migration in inflammatory responses and were shown to influence immune cell phenotypes.^28^, ^29^ Previous studies on embryonic, developing skeletal muscle and C2C12 myoblasts focused on the importance of VCAN processing by ADAMTS5, which was found at high levels during in vivo myogenesis around the stage of myoblast fusion to form myotubes.^27^ Here, we demonstrate only the up‐regulation of VCAN, without an increase in ADAMTS5 expression, suggesting the existence of a timely, highly coordinated strategy to support each stage of the regeneration process.

On the other hand, the genes down‐regulated at both experimental time‐points are generally associated with myogenic cells—LAMA2, TIMP3, and VTN. However, their levels were more drastically altered at 3 days post‐injury, the time‐point when myogenic differentiation starts. Previous studies on 2 different injury models (cardiotoxin and overload) reported that TIMP3 is constitutively expressed in mouse SCs and its swift but transient down‐regulation is required for myogenic differentiation and myotubes formation.^30^

Our results also showed a late induction (at 5 days post‐injury) of the alpha 1 chain of fibrillary collagen type III, V and VI as compared to internal control, most probably in interstitial muscle fibroblasts. This was consistent in both models of severe muscle damage, crush and freezing injuries, as opposed to an early and limited increase in the CI model. Besides the obvious mechanical role during skeletal muscle regeneration, collagen molecules have been shown to be essential constituents of the SC niche and critical for regulating SC behaviour during muscle regeneration when they form thick networks enclosing myogenic cells.^31^ However, late and excessive collagen production leading to muscle substitution may be more indicative of impaired functional recovery.

Our data mining approach proved that the expression profile of genes encoding various ECM and adhesion molecules varies according to the injury model, with only few genes being constantly up‐regulated. Transcript levels and time of induction may be influenced not only by the type of injury but also by its severity. For the genes tested in our panel, expression levels were significantly increased only at 3 days after eccentric contraction and only at 7 days after inflicting freeze injury. Our crush injury model has a different pattern, with an early and more complex induction of the cell‐to‐ECM adhesion molecules, more probably deriving from the invading myeloid cell population. The ECM protease profile, which was rather scarce, with a constant and strong up‐regulation of MMP8, is most probably also a consequence of the invading myeloid populations, along with the early onset of TIMP‐1 up‐regulation.^24^

Our crush model, similar to the FI model, inflicts a major injury as opposed to the eccentric contraction model analysed in the GSE5413 data set. This potentially explains the completely different profile for ECM and adhesion molecules. The up‐regulation of gene expression was higher, more diverse and with a later induction response (5 or 7 days) in the direct injury models (crushing and freezing, respectively), as opposed to the less severe injury induced by the contraction overload.

Consistent in all three injury models was the up‐regulation of Collagen Type III Alpha 1 Chain, the predominant form of collagen in the endomysium.^32^ Its induction time varies with the severity of the injury with later onset in the case of the more severe injuries, correlating with the late onset of MMP up‐regulation and matrix remodelling.

POSTN was the most up‐regulated gene in our model, much like in the FI model, and started to increase at a later time‐point than after the contractile overload of the CI model (which found it transiently changed at 3 days post‐injury). Our data suggest that delayed up‐regulation of POSTN may be indicative of direct destruction of muscle tissue and of the unfavourable outcome of ECM remodelling for architecture restoration. As demonstrated by the histologic analysis, inflammation persisted up to 14 days post‐injury and there were also signs of developing fibrotic scars, which suggested that the regeneration process was overwhelmed. POSTN has been recently shown to promote fibroblast migration at the injury site and to favour scar formation.^33^ POSTN was also previously reported to be transiently up‐regulated around 4 days post‐injury in a mouse model of cardiotoxin injury. The protein expression was first restricted to myoblasts and regenerating myofibres and then transferred to endomysial stromal cells, other than infiltrating myeloid cells.^19^

One limitation of this comparative study is the potential difference in regeneration responses among the different muscles of the calf (in our study the gastrocnemius muscle). Multiple reports suggest that the expression pattern of many of the tested genes depends on the type of muscle and the phase of muscle regeneration.^25^ Thus, a potential source of inaccuracy when comparing the data sets may be the different secondary time‐point post‐injury which in our case was at 5 days. However, in our view, this time‐point is a more accurate window into the early stage of muscle regeneration.

Another source of imprecision could be the potential effect on gene expression levels of the ketamine anaesthesia that was not performed on the control group nor by the other studies. This was at least partially mitigated by performing the injection in a different leg to the one receiving muscle injury.

## 5 CONCLUSION

In conclusion, our study revealed remarkable changes in gene expression profile for specific ECM and adhesion molecules during first stages of skeletal muscle regeneration after acute crush injury. Most of the genes for cell‐to‐ECM adhesion molecules as well as MMPs and protease inhibitors could represent the hallmark of myeloid cell population. The profile varied according to the experimental model and the severity of the injury influenced transcript levels and their induction time, as revealed by comparing our data with previous analyses on different injury models. The up‐regulation of gene expression was higher, more diverse and with a later induction following more severe trauma. The most up‐regulated gene was POSTN, which may be indicative of severe muscle damage and unfavourable architecture restoration. However, further studies testing the corresponding protein expression would bring more conclusive data to clarify their role in muscle regeneration. Moreover, based on these results, the intervention of various cell types during early stages of tissue regeneration after acute trauma could be further dissected and clarified.

## 6 CONFLICT OF INTEREST STATEMENT

The authors declare that they have no competing interests.

## AUTHOR CONTRIBUTION

**Laura Cristina Cristina Ceafalan:** Conceptualization (lead); Formal analysis (equal); Investigation (equal); Methodology (equal); Project administration (equal); Resources (equal); Supervision (equal); Validation (equal); Visualization (equal); Writing‐original draft (lead); Writing‐review & editing (equal). **Maria Dobre:** Data curation (equal); Formal analysis (equal); Investigation (equal); Methodology (equal); Visualization (supporting); Writing‐review & editing (supporting). **Elena Milanesi:** Data curation (equal); Formal analysis (equal); Investigation (equal); Methodology (equal); Visualization (equal); Writing‐original draft (supporting); Writing‐review & editing (supporting). **Andrei Marian Niculae:** Investigation (supporting); Methodology (supporting); Visualization (supporting); Writing‐review & editing (supporting). **Emilia Manole:** Investigation (supporting); Methodology (supporting); Visualization (supporting); Writing‐review & editing (supporting). **Mihaela Gherghiceanu:** Investigation (supporting); Methodology (supporting); Visualization (equal); Writing‐review & editing (supporting). **Mihail Eugen Hinescu:** Funding acquisition (lead); Project administration (equal); Supervision (equal); Validation (equal); Writing‐review & editing (lead).

## Supporting information


**File S1**.

**File S2**.

**File S3**.

## Data Availability

All the new data generated or analysed during this study are included in this published article as File S1. The analysis reported in the File S3 refers to Data Set GSE5413 in the Gene Expression Omnibus Database ^14^ https://www.ncbi.nlm.nih.gov/geo/query/acc.cgi?acc=GSE5413
